# Central auditory maturation and behavioral outcomes after cochlear implantation in prelingual auditory neuropathy spectrum disorder related to *OTOF* variants (DFNB9): Lessons from pilot study

**DOI:** 10.1371/journal.pone.0252717

**Published:** 2021-06-07

**Authors:** Sang-Yeon Lee, Jin Hee Han, Hoo-Kang Song, Namju Justin Kim, Nayoung Yi, Jeong-Sug Kyong, Byung Yoon Choi

**Affiliations:** 1 Department of Otorhinolaryngology-Head and Neck Surgery, Seoul National University Hospital, Seoul, South Korea; 2 Department of Otorhinolaryngology-Head and Neck Surgery, Seoul National University Bundang Hospital, Seongnam, South Korea; 3 Department of Audiology and Speech Language Pathology, HUGS Center for Hearing and Speech Research, Hallym University of Graduate Studies, Seoul, South Korea; 4 Department of Biological Sciences, Vanderbilt University, Nashville, Tennessee, United States of America; 5 Department of Otorhinolaryngology-Head and Neck Surgery, Chungnam National University College of Medicine, Daejeon, Korea; University of California, Los Angeles, UNITED STATES

## Abstract

The cortical auditory evoked potential (CAEP)-based P1 component acts as a biomarker for cochlear implantation (CI) outcomes in children with auditory neuropathy spectrum disorder (ANSD). To date, early intervention primarily before the age of two years and six months of CI usage is necessary and sufficient to achieve age-appropriate cortical maturation and good prognosis. However, varying degrees of neural dyssynchrony, resulting from the etiological heterogeneity of ANSD, may preclude uniform application of this hypothesis to ensure auditory cortical maturation. Thus, a focused evaluation of those carrying *OTOF* variants, which may be the salient molecular etiology of prelingual ANSD, would circumvent the issue of heterogeneity. Here, we sought to provide a much better understanding of the brain perspectives (i.e., P1 maturation) in *OTOF*-associated ANSD subjects and set the stage for an optimal strategy to enhance language development. We conducted a preliminary study comprising 10 subjects diagnosed with *OTOF*-related ANSD who underwent CI by a single surgeon and subsequently underwent measurements of the P1 component. We observed that DFNB9 subjects who received CI after 2 years of age exhibited “absent” or “anomalous” P1 components that correspond to delayed language development. However, timely implantation, as early as 12 months of age *per se*, might be insufficient to achieve age-appropriate cortical maturation of DFNB9 in cases with six to seven months of device use. This suggests the importance of sustained rehabilitation in DFNB9 than in other etiologies. Indeed, an additional follow-up study showed that a reduction in P1 latency was linked to an improvement in auditory performance. Collectively, our results suggest that central auditory maturation and successful outcome of CI in DFNB9 may have more demanding requirements, that is, earlier implantation and more sustained rehabilitation. We believe that the current study opens a new path toward genome-based neuroimaging in the field of hearing research.

## Introduction

Auditory neuropathy spectrum disorder (ANSD) is a specific hearing condition characterized by impaired neural transmission of sound signals from the inner ear to the auditory cortex, with normally functioning outer hair cells [1–3]. Although individuals with such a neural pathognomonic deficit in ANSD (i.e., dyssynchrony) respond to many sounds appropriately, they may experience impaired speech and language decoding, particularly in the area of temporal processing [4]. Clinically, subjects with ANSD manifest distinct auditory phenotypes, such as the presence of otoacoustic emissions (OAEs) or robust cochlear microphonics (CM), which are inverted with reversal of the polarity of the stimulus, and the absence or severe impairment of the auditory brainstem response (ABR) [5]. ANSD is relatively common, affecting 10–15% of subjects with sensorineural hearing loss worldwide. Theoretically, a presynaptic type of ANSD, in which the pathologic lesions lie peripheral to the spiral ganglion neuron (SGN), is expected to yield favorable cochlear implantation (CI) outcomes, because CI directly stimulates the SGN. In contrast, we can reason that the prognosis of CI among post-synaptic ANSD subjects with a main pathology, either in the SGNs or more central to SGNs (i.e., central auditory tract), would be not as favorable as that of CI among presynaptic ANSD subjects. Molecular genetic testing helps to determine whether subjects have post-synaptic or presynaptic ANSD. Indeed, genetic causes account for approximately 50% of these cases [6, 7].

Recent studies have shown a higher genetic load of *OTOF* variants, accounting for 91% of prelingual ANSD cases with an anatomically intact cochlear nerve (CN) [6]. *OTOF* that encodes otoferlin causes a recessive profound prelingual deafness (DFNB9) when mutated [8–10]. The audiological phenotype in DFNB9 patients and otoferlin-defective mice shows either absence or severe impairment of ABRs and preservation of OAEs, which clearly indicates auditory neuropathy [11]. Otoferlin localizes to the ribbon synaptic vesicles primarily in the cochlear inner hair cells (IHCs), and it is essential for calcium-dependent synaptic vesicle exocytosis via ribbon synapses [11, 12]. Furthermore, it has been suggested that defective otoferlin in DFNB9 may impede vesicle recycling and membrane trafficking [13]. Given that presynaptic lesions are mainly involved in DFNB9, children with *OTOF* variants significantly benefit from CI, achieving nearly peak speech perception scores postoperatively [9, 14].

Importantly, cortical auditory evoked potential (CAEP) can reveal a state of age-appropriate cortical maturation, which potentially correlates with language development in pediatric patients with deafness [15]. It is believed that identifying the P1 component, generated by repetitive excitatory input signals from the thalamus to the auditory cortex (i.e., lateral portion of Heschl’s gyrus, layers II–VI), may allow an objective measure to predict auditory cortical maturation and confirm successful auditory rehabilitation through CI [16]. Similarly, cortical event-related potentials (ERPs), including the P1 component, can be recorded in children with ANSD and can be used to predict speech perception ability and rehabilitation benefits [17]. Specifically, a previous study showed that there was a stark contrast in auditory cortical maturation between ANSD subjects who underwent CI before and after the age of two years. The former led to a normal P1 component in over 70% of cases, while the latter yielded delayed P1 latencies in the same proportion [17]. These results suggest that children with prelingual ANSD are more sensitive to age at intervention. Consistent with this, recent studies, including our previous studies, have demonstrated that for DFNB9, earlier intervention (i.e., before 2 years of age), when compared with other genetic deafness, could serve as an important determinant of favorable outcomes [6, 14]. This suggests that there is a limited time window for brain plasticity. Thus, early intervention within the sensitive period is hypothesized to be necessary for auditory cortical maturation in DFNB9 subjects.

Typically, early intervention, that is, before the age of two years, as well as CI usage for six months in patients with ANSD, have been reported to achieve age-appropriate cortical maturation and good prognosis [17]. A longitudinal follow-up study by Cardon et al. showed that the P1 component in most children with ANSD markedly improved six months after CI [17]. However, this duration is not collectively applicable in predicting central auditory development in all prelingual ANSD subjects due to varying degrees of neural dyssynchrony that result from the etiological heterogeneity of ANSD [18, 19]. Thus, studying one specific ANSD subgroup (those carrying OTOF variants) may help to control for heterogeneity, although there may still be inter-subject differences with respect to the severity, deafness duration, and other relevant factors (e.g., genotype) [8, 20].

Intriguingly, a recent electrophysiological study demonstrated that cochlear implantees with *OTOF* variants revealed significantly delayed ‘postsynaptic’ or more central neurotransmission with the device on, despite the presumed mechanism of DFNB9 involving ‘presynaptic’ transmission between hair cells and SGNs [21]. Additionally, the widespread expression of otoferlin, including in the brain, and its association subcellularly with the endosomal trans-Golgi network, may suggest a more ubiquitous role, likely influencing the development of both the central and peripheral auditory systems [22]. This, in turn, led us to hypothesize that the delayed or impaired auditory cortical maturation of unrehabilitated DFNB9 subjects could be attributed to both presynaptic and yet-to-be-identified post-synaptic pathology. To the best of our knowledge, auditory cortical maturation following CI has never been explored exclusively in DFNB9 subjects.

In this study, we aimed to unveil any distinct characteristics of OTOF-associated ANSD subjects (DFNB9) in the context of central auditory development. We hypothesized, at the least, that normal language development in those who receive earlier intervention and sufficient rehabilitation experience correlates with normal cortical maturation, whereas delayed cortical maturation may be associated with late intervention, insufficient use of the device, or a combination of both. In this pilot study, we evaluated the P1 component with reference to age at CI (< 2 years vs. > 2 years) and experience with device use (> 1 year vs. < 1 year), and we performed a longitudinal follow-up study of the P1 component in DFNB9 subjects with minimal experience of device use (6–7 months). Overall, as used here, using a combined approach, integrating genomics with neuroimaging, may not only provide a much better understanding of brain perspectives in *OTOF*-associated ANSD patients, but also set the stage for developing an optimal strategy for better language development.

## Materials and methods

### Participants

We conducted a retrospective review of 10 subjects diagnosed with *OTOF*-related DFNB9, who underwent CI by a single surgeon (B.Y.C.) and received measurements of the P1 component. Electrophysiological tests showed absent ABR with preservation of OAEs or CMs in all subjects, confirming the diagnosis of bilateral ANSD. Neither CN deficiency nor brain abnormalities were observed through the neuroradiological evaluation prior to CI. None of the subjects had a history of brain surgery, head injury, or neurological disorders that may hinder reliable P1 testing. This study was approved by the Seoul National University Bundang Hospital (SNUBH) Institutional Review Board (IRB No. B-2007-622-114) and was conducted in accordance with the principles of the Declaration of Helsinki. Due to the retrospective nature of this study, SNUBH IRB waived the requirement of informed consent.

### Demographics and clinical characteristics

As proposed by our hypothesis, CI before and after the age of two years were classified as “early intervention” and “late intervention,” respectively. Additionally, an individual’s experience with device use was defined as the time interval between turning on the CI and postoperative P1 testing. Typically, ANSD patients with various underlying etiologies who receive early implantation before the age of two years have also been shown to have normalized age-appropriate P1 waves at approximately six months postoperatively [17]. Considering this, we randomly hypothesized that one year would be sufficient to induce normal P1 waves, as this duration is twice as long as six months. Given this, our cohort was further classified into those with “sufficient experience” (i.e. > 1 year) and “insufficient experience” (i.e. < 1 year) to identify the stark difference in P1 maturation, depending on an individual’s experience with device use. In our cohort, three subjects showed a remarkably short experience with their device (approximately six to seven months), while the remaining subjects had relatively long experience with their device (more than two years). Based on our criteria, subjects were divided into three groups: early intervention with sufficient experience (group 1, N = 3), early intervention with insufficient experience (group 2, N = 3), and late intervention with sufficient experience (group 3, N = 4) (Table 1). The median age at CI was 12 months for both groups 1 and 2 (range, 10–14 months) and 36 months for group 3 (range, 25–400 months). The average follow-up duration between CI and CAEP testing for groups 1, 2, and 3 was 36 months (range, 36–52 months), 7 months (range, 6–10 months), and 82 months (range, 26–100 months), respectively. All subjects in groups 1 and 2, as well as two of the four subjects in group 3, underwent bilateral simultaneous CI, whereas the remaining two subjects in group 3 underwent bilateral sequential CI. Moreover, there was no difference in the preoperative hearing status (unaided and aided) between the two adult subjects (Subjects 9 and 10) for which behavioral audiometry was possible.

**Table 1:** Demographics and clinical characteristics of 10 subjects with DFNB9.

Group	Subject	Age at CI	Sex	CI Strategy	CI Experience	Baseline evaluation (Preoperative S/E)		Postoperative evaluation					
								CAEP (postoperative initial)			Postoperative S/E		
						CAP	IT-MAIS	Age at evaluation	P1 latency	Z-score	CAP	IT-MAIS	SIR
**group 1**	1	10mo	M	bilateral simultaneous	4Y4mo	0	4	5Y2mo	Normal	0.47	7	40	5
	2	1Y2mo	F	bilateral simultaneous	3Y	1	0	4Y2mo	Normal	0.48	5	40	4
	3	1Y2mo	M	bilateral simultaneous	3Y	1	0	4Y2mo	Normal	1.39	5	40	4
**group 2**	4	1Y	F	bilateral simultaneous	6mo	0	9	1Y6mo	delayed	2.76	4	32	2
	5	1Y2mo	M	bilateral simultaneous	7mo	1	9	1Y9mo*	delayed	2.35	4	36	2
	6	1Y	M	bilateral simultaneous	6mo	1	11	1Y6mo*	delayed	2.35	4	38	2
**group 3**	7	3Y	M	bilateral sequential	2Y2mo	0	1	5Y2mo	Normal	0.81	4	36	3
	8	3Y1mo	M	bilateral simultaneous	6Y1mo	0	6	9Y2mo	Absent	4	3	37	2
	9	25Y	F	bilateral sequential	11Y6mo	0	NA	36Y6mo	Normal	0.45	5	NA	4
	10	27Y	M	bilateral sequential	8Y4mo	0	NA	35Y4mo	Absent	4	NA	NA	NA

Abbreviation: ANSD, auditory neuropathy spectrum disorder; CI, cochlear implantation; S/E, speech evaluation; CAEP, cortical auditory evoked potential; CAP, categorization of auditory performance; IT-MAIS, Infant-Toddler Meaningful Auditory Integration Scale; SIR, speech intelligibility ratio; Y, year; mo, month; M, male; F, female; NA, not available.

* Note that the two subjects (subject No. 5 and 6) in group 2 were available to evaluate the additional P1 measurement at 5 months after their initial evaluation. Their follow-up CAEP testing was performed at 11 and 12 months, respectively, after cochlear implantation.

### Molecular genetic testing

A real-time PCR-based new kit (U-TOP^™^ HL Genotyping Kit Ver2) using the Melting Array technique was initially tested against the five variants from *OTOF* (Glu841Lys, Arg1856Trp, Leu1011Pro, Tyr1064Ter, and Arg1939Gln) in subjects manifesting ANSD phenotype [23]. The *OTOF* variants (Glu841Lys, Arg1856Trp, Leu1011Pro, Tyr1064Ter, and Arg1939Gln) were highly prevalent among Korean subjects with prelingual ANSD [6, 20, 24]. Subsequently, exome sequencing was performed to verify that the subjects did not carry convincing variants in the deafness panel, followed by bioinformatics analyses, as described previously [25, 26]. The resulting readings were compared to the UCSC hg19 reference genome, and non-synonymous SNPs were filtered with a depth = 40; dbSNP138 was filtered out, except for the flagged SNP. Using a comprehensive filtering process, rare single-nucleotide variations, indels, or splice-site variants were chosen, as described in our previous studies [25, 26]. Subsequently, Sanger sequencing and segregation analyses were used to confirm the variant of the candidate deafness genes. To predict the pathogenic potential of each detected variant, in silico studies using SIFT (http://sift.jcvi.org/) and PolyPhen2 (http://genetics.bwh.harvard.edu/pph2/) were performed. Additionally, the GERP++ score from the UCSC Genome Browser (http://genome.ucsc.edu/) was used to determine the evolutionary conservation of the amino acid residues.

After analytical step and filtering strategy, Sanger sequencing and segregation analyses confirmed the causative *OTOF* variants responsible for DFNB9 (Table 2). Specifically, Subject 4 turned out to carry only a single heterozygous OTOF variant; however, we strongly believe that this subject has DFNB9. First, subject 4 has ANSD without any anatomical defect in the cochlear nerve, which had been previously reported to have a homogeneous molecular etiology of DFNB9 [6]. Further, the detected variant of OTOF in this subject is considered ’pathogenic’, making it least likely that this variant is incidentally detected in this ANSD subject. The trans allele to the detected variant is presumed to either contain a structural variant or quantitatively affects expression of otoferlin.

**Table 2:** Mutational spectrum of subjects with DFNB9 in our cohort.

Family ID	Variant (*OTOF*) NM_001287489: NP_001274418 dbSNP ID	State	Prediction Algorithm			Conservation Score		MAF		Classification of pathogenic variants
			Mutation Taster	PolyPhen-2	SIFT	PhyloP	GERP++	Global MAF	KRGDB (n = 1722)	
group 1										
Subject 1	c.5816G>A: p.Arg1939Gln	Het	DC	PD	D	2.261	2.28	T = 0.00003/1 (ExAC)	T = 0.001452/5	Pathogenic
	rs201326023							T = 0.0002/1 (1000 Genomes)		
	c.5566C>T: p.Arg1856Trp	Het	DC	PD	D	2.963	2.84	A = 0.00004/5 (ExAC)	A = 0.000871/3	Pathogenic
	rs368155547							A = 0.00008/1 (GO-ESP)		
Subject 2	c.2521G>A: p.Glu841Lys	Het	DC	PD	D	5.523	5	T = 0.00003/3 (ExAC)	ND	Pathogenic
	rs772729658									
	c.3032T>C: p.Leu1011Pro	Het	DC	PD	D	5.012	4.64	ND	ND	Pathogenic
	rs80356596									
Subject 3	c.5816G>A: p.Arg1939Gln	Het	DC	PD	D	2.261	2.28	T = 0.00003/1 (ExAC)	T = 0.001452/5	Pathogenic
	rs201326023							T = 0.0002/1 (1000 Genomes)		
	c.2521G>A: p.Glu841Lys	Het	DC	PD	D	5.523	5	T = 0.00003/3 (ExAC)	ND	Pathogenic
	rs772729658									
group 2										
Subject 4^a^	c.2521G>A: p.Glu841Lys	Het	DC	PD	D	5.523	5	T = 0.00003/3 (ExAC)	ND	Pathogenic
	rs772729658									
Subject 5	c.5816G>A: p.Arg1939Gln	Het	DC	PD	D	2.261	2.28	T = 0.00003/1 (ExAC)	T = 0.001452/5	Pathogenic
	rs201326023							T = 0.0002/1 (1000 Genomes)		
	c.2521G>A: p.Glu841Lys	Het	DC	PD	D	5.523	5	T = 0.00003/3 (ExAC)	ND	Pathogenic
	rs772729658									
Subject 6	c.5566C>T: p.Arg1856Trp	Het	DC	PD	D	2.963	2.84	A = 0.00004/5 (ExAC)	A = 0.000871/3	Pathogenic
	rs368155547							A = 0.00008/1 (GO-ESP)		
	c.2521G>A: p.Glu841Lys	Het	DC	PD	D	5.523	5	T = 0.00003/3 (ExAC)	ND	Pathogenic
	rs772729658									
group 3										
Subject 7	c.4227+5G>C	Het	DC	NA	NA	1.616	3.95	G = 0.00006/7 (ExAC)	ND	Pathogenic
	rs571671530							G = 0.0002/1 (1000 Genomes)		
	c.2521G>A: p.Glu841Lys	Het	DC	PD	D	5.523	5	T = 0.00003/3 (ExAC)	ND	Pathogenic
	rs772729658									
Subject 8	c.3192C>G:p.Tyr1064Ter	Het	DC	NA	NA	0.951	2.78	C = 0.000004 (1/250556, GnomAD_exome)	C = 0.000292/1	Pathogenic
	rs766819324									
								C = 0.000008 (1/119874, ExAC)		
	c.5203del:p.Arg1735Glyfs*28	Het	DC	NA	NA	1.688	4.17	delG = 0.000004 (1/251374, GnomAD_exome)	ND	Pathogenic
	rs727503352							delG = 0.000008 (1/121144, ExAC)		
Subject 9	c.5816G>A: p.Arg1939Gln	Het	DC	PD	D	2.261	2.28	T = 0.00003/1 (ExAC)	T = 0.001452/5	Pathogenic
	rs201326023							T = 0.0002/1 (1000 Genomes)		
	c.3032T>C: p.Leu1011Pro	Het	DC	PD	D	5.012	4.64	ND	ND	Pathogenic
	rs80356596									
Subject 10	c.5816G>A: p.Arg1939Gln	Het	DC	PD	D	2.261	2.28	T = 0.00003/1 (ExAC)	T = 0.001452/5	Pathogenic
	rs201326023							T = 0.0002/1 (1000 Genomes)		
	c.2521G>A: p.Glu841Lys	Het	DC	PD	D	5.523	5	T = 0.00003/3 (ExAC)	ND	Pathogenic
	rs772729658									

Abbreviation: Het, heterozygote variant; DC, disease causing; PD, probable damaging; D, damaging; ND, not detected; NA, not applicable.

PhyloP score from the Mutation Taster (http://www.mutationtaster.org/).

in silico prediction Algorithm: Polyphen-2 (http://genetics.bwh.harvard.edu/pph2/index.shtml);

SIFT (http://sift.jcvi.org/www/SIFT_chr_coords_submit.html) or SIFT-indels2 (http://sift.bii.a-star.edu.sg/www/SIFT_indels2.html);

Conservation tools: GERP++ score in the UCSC Genome Browser (http://genome-asia.ucsc.edu/);

ExAC, Exome Aggregation Consortium (http://exac.broadinstitute.org/).

gnomAD: The Genome Aggregation Database (https://gnomad.broadinstitute.org/).

1000 Genomes (https://www.ncbi.nlm.nih.gov/variation/tools/1000genomes/).

KRGDB, Korean Reference Genome DB (http://152.99.75.168/KRGDB/).

^a^ Note that the remaining allele is considered a structural variant or allele that influences expression.

### EEG recording and stimuli P1 component

Throughout the recording, participants were seated comfortably in a chair or on their caregiver’s lap in a sound-attenuated booth. EEG recordings were made using a Geodesic Sensor Net with 64 electrodes (FP1, FPz, FP2, AF3, AF4, F7, F5, F3, F1, Fz, F2, F4, F6, F8, FT7, FC5, FC3, FC1, FCz, FC2, FC4, FC6, FT8, T9, T7, C5, C3, C1, Cz, C2, C4, C6, T8, T10, TP9, TP7, CP5, CP1, CP2, CP6, TP8, TP10, P9, P7, P5, P3, P1, Pz, P2, P4, P6, P8, P10, PO3, POz, PO4, O1, Oz, and O2) electrodes (Philips, Electrical Geodesic, Inc., Eugene, OR, USA). An additional four channels were tagged as electro-oculograms to record eye-movement data. Impedances were kept below 100 kΩ throughout the recording period. EEG data were 0.1–220 Hz band-pass filtered online at a sampling rate of 1000 Hz. CAEPs were evoked using a 90-milliseconds (ms) /ba/ sound recorded by a female speaker. The sound was presented at 65 dB SPL via loudspeakers located 45 °to the left and right ears, at an interval of 610 ms. Since we lacked baseline CAEP, and thereby intended to compare our patient’s auditory cortical maturity with the normative data by Campbell et al. (2011), we conformed strictly to the type of stimulus and the intensity level (65 dB SL, 90 ms long /ba/ sound) provided by Campbell et al. (2011) [27]. Additionally, in this study, DFNB9 children with *OTOF* variants were given a sufficiently high threshold phase-amplitude stimulus (65 dB SPL) with the CI device on, precluding inaudibility as a significant issue. Five formants were used for the stimulus. The starting frequencies of F1 and F2 were approximately 230 Hz and 605 Hz, respectively. The center frequencies of the vowel /a/ were 740, 2790, 3500, and 4600 Hz for F1, F2, F3, F4, and F5, respectively [28], while watching a silent video clip on a computer monitor [29]. A total of 200 sweeps were collected in each session, and this was repeated for replication within half an hour. The number of sweeps we employed (200 sweeps) may not be sufficient, especially in patients with ANSD. However, our sweep number fell within the range of the sweep number found in the literature, ranging from 30 [27] to 500 [30]. Our sweep number also corresponded to a value greater than 100 sweeps, as suggested by the British Society of Audiology [31].

Specifically, for the two subjects in group 2, the recording was conducted in two sessions, which were 5 months apart. Off-line processing of the EEG data included bad channel replacement, band-pass filtering (1–50 Hz), and baseline correction (− -100-0 ms). Eye blinks and facial movements-related artifacts were rejectedusing independent component (IC) analysis with additional visual inspection. The signals were then averaged from 100 ms before stimulus onset to 500 ms after onset of the stimuli. All the data were re-referenced to common averages. Additional artifacts associated with the CI device were minimized using previously described procedures from the literature [32] and from our laboratory. Specifically, ICs representing CI-associated artifacts were initially identified based on temporal and spatial characteristics; the ICs and residual artifacts were further rejected based on the two criteria of the IC waveform morphology and topography. We rejected the ICs containing a centroid of the activity around the CI in topography, as well as the ICs showing onset activity at the sound stimuli and an offset at the stimuli. The mean surviving epoch was 368 ± 16 (312–373) after rejecting the eye- or CI device-related artifacts (3–6 ICs in each run). Waveforms were averaged to create a grand average waveform for each subject. Signal processing was conducted using Matlab software (Mathworks, Natick, Massachusetts, USA), with toolboxes provided by Fieldtrip [33] and EEGLAB [34].

### P1 component

Cleaned data were pruned to the amplitudes and latencies of the P1 peak. P1 latency was calculated from the grand average waveform. The P1 component is characterized as the first positive curve preceding a large negative wave, namely, N1. The reliability of the P1 peak between the two sessions was further confirmed by the agreement between two audiologists, as described by Campbell et al. (2011) [27]. To assess the current neurophysiological status of our subjects against the normal development of P1 latencies, we referenced the data of 190 normal hearing subjects, ranging in age from 0.1 to 12 years [35–37]. For the P1 latencies in those aged over 12 years, the linear growth function of the best-fit line, based on the data in Sharma et al., was applied to estimate P1 latency [33, 34]. In this study, the individual Z-score was calculated based on the growth function of the best-fit line that stems from a predictive model obtained from previous reports consisting of the largest number of subjects thus far, which positively correlates with the P1 latency [16, 38]. Specifically, the values for subjects with “absent” P1 were converted based on the mean +4 standard deviation (SD), which denotes a nearly impossible occurrence in a small data set [39].


Expectedlatency=155.6+(−32.746*ln(age))

### Statistical analyses

All statistical analyses were performed using R statistical software (R version 3.5.2, R Studio 1.0.136; Foundation for Statistical Computing, Vienna, Austria). All analyses were performed using GraphPad Prism version 8.0.0, for Windows (GraphPad Software, San Diego, California, USA; www.graphpad.com). All data are presented as mean ± standard error of the mean (SEM). To analyze the relationships between P1 latency (i.e., individual Z-score) and behavioral outcomes (postoperative categorization of auditory performance [CAP], Infant-Toddler Meaningful Auditory Integration Scale [IT-MAIS], and speech intelligibility ratio [SIR] at time point for CAEP), Pearson’s correlation analyses were performed, because the Z-score, CAP, SIR, IT-MAIS, and follow-up period were normally distributed. Furthermore, a correlation analysis between P1 latency (i.e., individual Z-score) and follow-up periods (i.e., CI usage) in those who received CI before 2 years of age was performed. Statistical significance was set at p < 0.05.

## Results

### Variations in P1 component between groups

**Fig 1:**
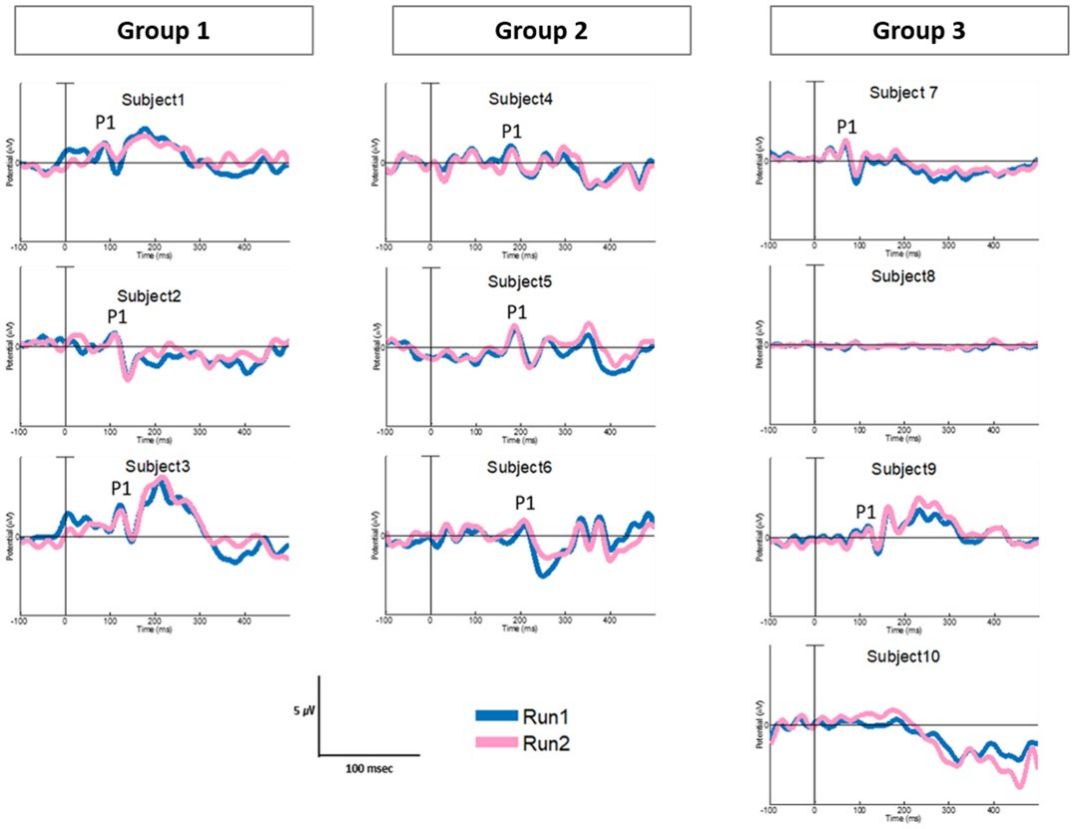
Individual cortical auditory-evoked potential in groups 1, 2, and 3. Each patient in all groups showed typical P1 with varying latencies, except the last two patients in group 3, who displayed anomalous (absent) P1 morphology.

**Fig 2:**
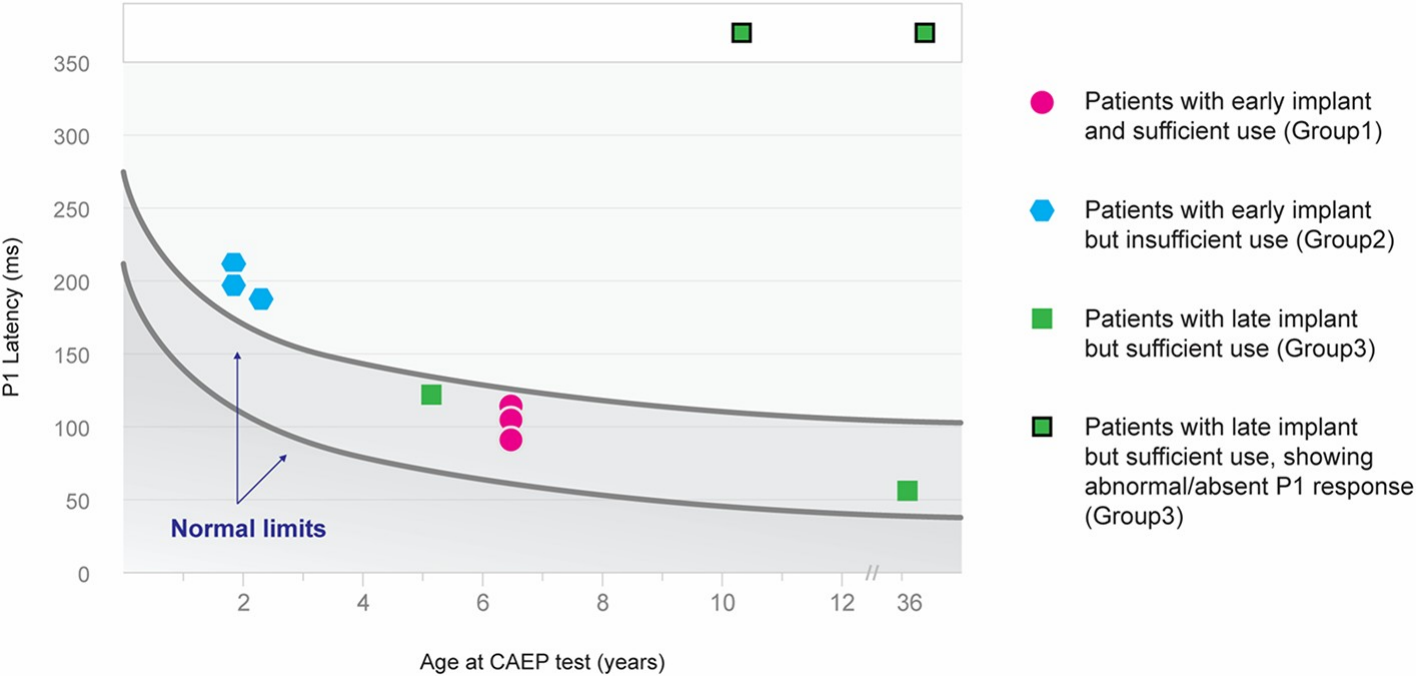
P1 latency as a function of age for 10 patients fit with CI. Circles indicate latencies in the patients within the normal P1 range after sufficient use with early (pink circles) or late (green squares with no border line) implantation. Hexagons indicate latencies in patients with delayed P1 response who had insufficient use of CI in spite of early stimulation. Green squares with black borderline indicate latencies in the patients with late implantation and insufficient use resulting in abnormal/absent P1 response. The upper and lower solid lines indicate the 95% confidence intervals for the 190 normal-hearing data (Sharma et al. 2002a).

Fig 1 illustrates the individual CAEP in the three groups. The P1 component showed distinct characteristics in terms of latency and presence in the three groups. All subjects in group 1 exhibited typical morphology consisting of reproducible and robust P1 peaks in the waveforms and P1 latencies that fell within the 95% confidence intervals for normal P1 latency development (Fig 2). This suggests that early intervention, before the sensitive period, and sufficient rehabilitation allowed for normal cortical maturation of the central auditory system. Conversely, all subjects in group 2 showed reproducible and robust P1 peak waveforms, but markedly “delayed” P1 latencies than those in group 1 (Fig 2). The “delayed” P1 latencies presented in group 2 fell outside the 95% confidence intervals for normal P1 latency development, suggesting that the device experience of 6 months, regardless of the early intervention at an average age of 1 year, may not be sufficient to catch up to age-appropriate synchronization of neural transmission and normal auditory cortical maturation. Meanwhile, two subjects in group 3 (subject No. 7 and 9) displayed typical P1 components with age-appropriate latencies, while the remaining two (subject No. 8 and 10) consistently illustrated “absent” or “anomalous” P1 components, despite of sufficient CI experience of more than 2 years (Fig 2). Expectedly, our preliminary study observed that DFNB9 subjects who received the CI after 2 years of age tended to exhibit “absent” or “anomalous” P1 components that correspond to delayed language development. This requires further confirmation because of the small sample size of this study.

### Longitudinal analysis on the P1 component

Fueled by P1 latency not yet normalized even after at least 6 months of device use in group 2, we aimed to determine how long it would take for the P1 latency in such early implantees with DFNB9 to enter the normal range. To that end, we performed an additional follow-up study investigating the P1 component trajectory for DFNB9 subjects with short-term CI experience (6–7 months). Two subjects (subject No. 5 and 6) in group 2 were included in this additional P1 measurement at 5 months after their baseline evaluation, making their follow-up CAEP testing at 11 and 12 months, respectively, after CI.

**Fig 3:**
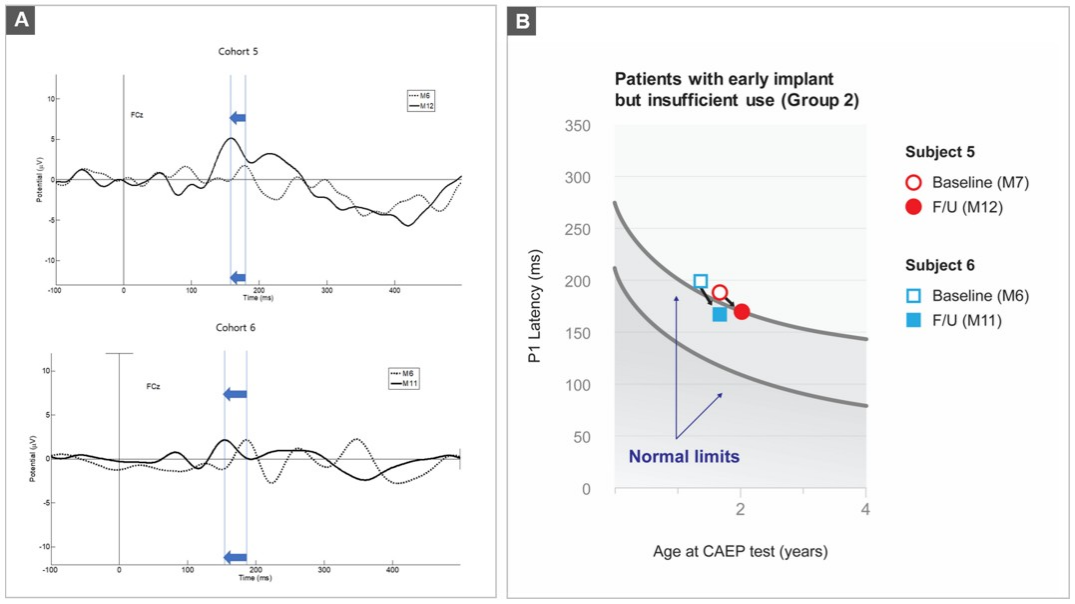
Longitudinal observation of the P1 latency in subjects 5 and 6. (A) A decrease of 23 msec in subject 5 and a decrease of 38 msec in subject 6 during the follow-up test. (B) The trajectories of P1 latency superimposed on the line of best-fit with the 95% confidence interval for the 190 normal-hearing listeners (Sharma et al. 2002a). The empty symbols indicate the first test, and the filled symbols indicate the second test.

Compared to the baseline, the latency of P1 markedly decreased over time, while the amplitude did not show any uniform pattern during the follow-up evaluations (Fig 3A). As a result, the P1 latencies of subjects No. 5 and 6 completely fell and almost fell (although across the borderline) within the 95% confidence intervals for normal P1 latency development, respectively (Fig 3B). Furthermore, the decrease in P1 latencies for both subjects was linked to an improvement in their auditory performance scores. These results suggest that sustained auditory rehabilitation for at least 1 year following CI may be an essential prerequisite for age-appropriate auditory cortical maturation of DFNB9 subjects from the perspective of the brain.

### Correlation of the P1 component and postoperative speech performance

**Fig 4:**
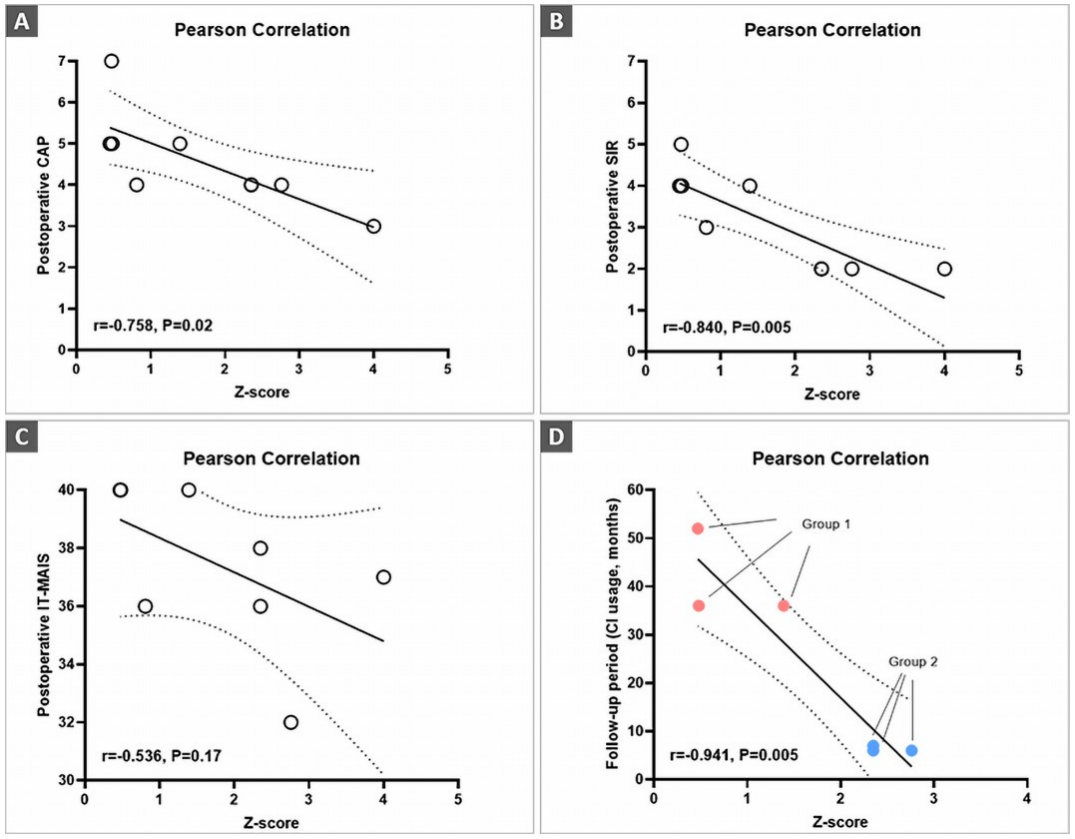
Correlation analyses of the auditory performance and P1 latency. (A, B) The Z-scores were found to be inversely correlated with preoperative CAP (r = -0.758, P = 0.02) and SIR (r = -0.840, P = 0.005). (C) A better Z-score as reflected by shorter P1 latency did not correlate with a higher postoperative IT-MAIS score, although a very weak tendency was observed (r = -0.536, P = 0.17). (D) The Z-scores were found to be inversely correlated with the follow-up duration between CI and CAEP testing, observed only in subjects included in groups 1 and 2 (r = -0.944, P = 0.005). The grey dotted color indicates the 95% confidence interval.

Auditory performance scores, including categorization of auditory performance (CAP), speech intelligibility rate (SIR), and IT-MAIS, were assessed. The speech was evaluated for postoperative auditory performance within 2 weeks of P1 testing. Correlation analyses revealed that a reduction in Z-scores tended to result in better postoperative CAP and SIR scores (r = -0.758, P = 0.02, and r = -0.840, P = 0.005, respectively), demonstrating that a reduction in P1 latency is associated with enhanced auditory performance (Fig 4A and 4B). As depicted in Fig 4C, a better Z-score as reflected by shorter P1 latency did not correlate with a higher postoperative IT-MAIS score, although a very weak tendency was observed (r = -0.536, P = 0.17). Our data are slightly different from those of previous reports, demonstrating a significant correlation between P1 latency and auditory skill development as measured by the IT-MAIS, most likely due to the smaller number of cases and methodological differences (e.g., evaluation timing). Alternatively, the ceiling effect of IT-MAIS scores due to parental expectations may not have a significant impact on the difference between P1 latency and auditory skill development as measured by the IT-MAIS, unlike findings observed in CAP and SIR scores. Furthermore, a significant inverse correlation between the Z-scores and the follow-up duration from CI to CAEP testing (i.e., CI usage) was observed only in groups 1 and 2 (r = -0.944, P = 0.005). Hence, sufficient device experience in DFNB9 subjects may be needed for normal auditory cortical maturation, even with early intervention (< 2 years of age) (Fig 4D), although the small sample size warrants further investigation.

## Discussion

To the best of our knowledge, this is the first pilot study to reveal the influence of ‘age at CI’ and the ‘duration of device use’ on auditory cortical maturation among genetically characterized DFNB9 subjects. In this study, we evaluated the P1 component with reference to age at the time of CI (<2 years vs. >2 years) and duration of device use (>1 year vs. <1 year). Furthermore, we performed a longitudinal follow-up analysis of the P1 component in DFNB9 subjects with short-term device use (6–7 months). Consistent with previous reports from prelingual ANSD subjects, without targeted etiologies [17, 40, 41], we also observed that DFNB9 subjects who received CI after the age of 2 years tend to exhibit “absent” or “anomalous” P1 components coupled with delayed language development, if not always. Conversely, it could be that two individuals in group 3 (subjects 7 and 9) showing normal P1 waves might have had a less severe form of ANSD and/or received some level of auditory stimulation adequate for central maturation prior to implantation. Indeed, we previously reported that there was a difference in the degree of residual hearing, depending on the *OTOF* genotypes [9], and subject 7 with a splice site variant residing in +5G could have had a significant degree of normal splicing transcripts, leading to significant residual hearing. Likewise, c.3032T>C: p.Leu1011Pro from subject 9 (adult) could have been associated with better residual hearing during childhood, which could be validated due to the lack of audiograms during childhood. In contrast, it is easily conceivable that subject 8, carrying two truncation *OTOF* alleles, could have a much more severe degree of ANSD. In addition, our study also revealed that timely implantation, as early as 12 months of age, *per se*, is not sufficient for age-appropriate cortical maturation of *OTOF*-related ANSD with only 6 months of device use. This suggests the importance of sustained rehabilitation in DFNB9 than in other etiologies. However, as in a pilot study, the relatively small sample size and lack of preoperative evaluation of the P1 component could have potentially led to misinterpretation of our results, which await further investigation.

Previous studies have consistently reported a narrow sensitive period for central auditory development or speech outcome in subjects with *OTOF*-related DFNB9 [6, 14, 42]. Specifically, early intervention (i.e., <2 years of age) seemed to be more beneficial with respect to language development than late intervention (i.e., >2 years of age) [14, 42]. Furthermore, early implantation (age at CI ≤ 18 months) showed a greater degree of rapid speech improvement after CI in DFNB9 subjects than in late implantation [6]. Concordantly, we observed an “absent” or “anomalous” P1 component in 50% of our DFNB9 subjects who underwent CI after the age of 2 years, albeit with sufficient device experience. Typically, the high plasticity of the brain during this sensitive period tends to elicit robust synaptic connections and cortical phase synchronization. Thus, prolonged neural dys-synchrony, due to late intervention after this narrow sensitive period specific to DFNB9 subjects, could have led to deficits in cortical maturation [43, 44], leading to continued exhibition of an “absent” or “anomalous” P1 component. Alternatively, late intervention-induced cross-modal reorganization, whereby cortical regions of the deprived modalities become vulnerable to recruitment by the remaining intact sensory domains, such as the visual and somatosensory cortices, might hinder age-appropriate cortical maturation [45, 46]. However, compared with non-genetic deafness, such as *GJB2-* or *SLC26A4*-related deafness, shows greater tolerance to delayed implantation, as late as up to 35 months after CI, as evidenced by better speech performance [47]. Such observations convey that the sensitive period for neural plasticity significantly differs from gene to gene, favoring the former interpretation. Indeed, wave III and wave V latencies obtained from cochlear implantees carrying the *OTOF* variants were significantly longer than those carrying the *GJB2* or *SLC26A4* variants, suggesting that postsynaptic (central to the SGNs) activity, such as CN synchrony, was still disturbed after implantation in DFNB9 subjects [21]. It was proposed that the prolonged disturbance in postsynaptic synchrony could be caused by insufficient presynaptic stimulation and/or delayed maturation of the nervous system, including both pre- and post-synaptic neuronal networks [21]. Collectively, earlier intervention could be crucial to auditory cortical maturation and successful behavioral outcomes of CI in subjects with *OTOF* variants.

Interestingly, contrary to a previous study, timely intervention of DFNB9 *per se* was not sufficient for the recovery of P1 maturation within six months among prelingual ANSD patients in our study [17]. Indeed, children with *OTOF*-associated DFNB9 were unable to catch up with normal-hearing peers in terms of P1 latency with only six to seven months of device use in our study, even though implantation was performed as early as 12 months of age. This suggests that ANSD shows obvious heterogeneity, not only in the etiologies, but also in the amount of stimulus required for auditory cortical maturation. Recently, as evidenced by the electrically evoked ABR, *OTOF* variants were more closely associated with delayed postsynaptic neurotransmission [21]. The mechanism of peripheral neural dyssynchrony due to alteration of *OTOF*, which has been reported to be expressed exclusively in the IHC and affects the synapse between the IHCs and SGNs, can influence maturation of the auditory cortex requires further research. In our current study, with one full year of auditory rehabilitation after device switch-on, P1 latency of both DFNB9 children (group 2) had just returned to the normal range of P1 latency. Therefore, DFNB9 pathology appears to require sustained stimulation to restore abnormal auditory cortical maturation secondary to peripheral neural dyssynchrony. To be paraphrased, the intrinsic deficits in central auditory pathways due to *OTOF* variants would respond to the restoration of peripheral neural synchrony by CI-induced electrical stimulation rather slowly.

Given that most hearing-impaired children with CI before the age of 3.5 years were able to show age-appropriate P1 response within three to six months of device use [16, 36, 38], it seems apparent that auditory rehabilitation of ASND due to *OTOF* variants may require earlier implantation and more sustained rehabilitation. In a clinical setting, these results would provide insights into how otologists/pediatricians should deal with DFNB9 children to attain age-appropriate auditory cortical maturation. It has been widely accepted that the CAEP-based P1 component acts as a biomarker of CI outcomes in children with ANSD [17]. A review demonstrated that up to 27% of subjects with ANSD did not reach 30% of open-set speech perception scores despite receiving appropriate and sufficient electrical stimulation via CI. The P1 component evaluation may provide an answer to this enigma, otherwise unexplained. Similarly, CI outcomes in DFNB9 subjects can vary in the context of language development [48], even if the pathophysiological mechanisms and expression of otoferlin are primarily limited to the synaptic regions [49]. As depicted in Fig 4, the meaningful correlation between central auditory maturation and behavioral outcomes of CI makes it possible to use CAEP data as an additional biomarker for the prediction of postoperative language development in DFNB9 subjects. Based on this, we believe that an evaluation of the P1 component may allow timely intervention and precise auditory rehabilitation in DFNB9 children, although to some degree, the identification of genetic etiology itself may determine their fate.

It is worth noting that the severity of the underlying neural dyssynchrony possibly inherent to *OTOF* genotypes and acoustic amplification history prior to implantation could also affect cortical auditory development and behavioral outcomes, in addition to the impact of age at intervention and duration of device use in DFNB9 patients. The different P1 responses observed in group 3 support this argument. Severe auditory neural dyssynchrony can not only lead to an atypical pattern of cortical stimulation, but also negatively affect behavioral outcomes [50]. Importantly, the development of cortical maturation is dependent first on molecular genetics, then on sensory input, if the appropriate stimuli are provided at the right time [51]. Based on this, a focused evaluation of only those carrying *OTOF* variants (DFNB9 patients), as presented herein, would circumvent the issue of heterogeneity in terms of the severity of neural dyssynchrony. Moreover, whether acoustic amplification via hearing aids helps central auditory maturation remains controversial. Although acoustic amplification improves synchrony to some extent by improving temporal processing, such as phase locking, at the level of IHCs and CN [50], the fact that the underlying pathophysiology of *OTOF* is limited to the peripheral IHCs, the CN may weaken the implications of acoustic amplification history on developing cortical maturation in DFNB9 patients. Supporting this, Sharma et al. also concluded that simply amplifying acoustic signals seems to offer benefits for a subset of ANSD patients with mild levels of dyssynchrony [18], which could occur in the case of milder pathogenic potential of a certain OTOF variant, such as a splice site variant at a residue of +5G. Indeed, subject 7 was the only patient who carried a splice site variant of *OTOF*. In fact, at least in Koreans, this residual hearing issue related to *OTOF* genotypes does not impose a significant dilemma in interpreting our results, since the major *OTOF* allele of p.R1939Q was previously reported to show the poorest residual hearing [9].

This study may stand out in the current era of precision medicine by incorporating genomics and brain neuroimaging for ANSD research, potentially paving the way for a ‘new normal’ in the future of brain neuroimaging of hearing-impaired subjects. Nevertheless, several limitations of this study should be addressed in future studies. First, as in a pilot study, our results were limited by the small number of study subjects and co-factors potentially relevant to the P1 component. In this study, confounders were minimized, but not eliminated. In particular, it is more likely that confounding factors related to cortical maturation, such as the degree of ANSD severity and amplification history prior to implantation, may affect the variability in group 3. Furthermore, our current study was mostly designed to be a cross-sectional evaluation, which, along with the retrospective study design, may weaken the clinical implications of the results. Therefore, a longitudinal follow-up study that assesses a large cohort is warranted to validate our hypotheses. Second, we observed that 50% of DFNB9 subjects in group 3 reached the cortical auditory maturation corresponding to a normal limit for age-appropriate P1 latency, despite the CI beyond the sensitive period. Although the molecular and cellular mechanisms of hearing loss are supposed to be identical among the individuals in group 3, the synaptic structure and function that regulate the neuromodulatory circuits like learning and attention would be different both quantitatively and qualitatively. This, in turn, may cause a difference in P1 maturation [52, 53]. However, this requires further research. Additionally, we acknowledge that a time course of 5 months could cause an enormous change in P1 latency, specifically in the pediatric population (see Fig 3). The latencies of the two subjects decreased over the course of five months and entered the normal range estimated by Campbell et al. (2011) [27]. Future studies should consider this issue in order to make a more precise prediction of the time point entering the normal range after CI in this kind of group. Third, the mapping strategy of our cohorts was carried out in the same manner; however, the time for CI use was not specified in detail. Thus, efforts to minimize confounders that affect CI outcomes are needed to draw a firmer conclusion. Lastly, a growing body of evidence indicates the variability of developmental quotients in various domains among CI recipients postoperatively [54]. As such, further studies involving a thorough longitudinal assessment of the cognitive, motor, and social domains, together with CAEP-based P1 evaluation, are warranted.

## Conclusion

Taken together, our pilot study suggests that central auditory maturation and successful behavioral outcomes of CI, especially in subjects DFNB9 related ANSD, may have more demanding requirements characterized by earlier implantation and more sustained rehabilitation than other genetic deafness. Our results may coincide with those of previous reports, suggesting that the pathophysiological outcome of *OTOF* variants is not likely to be necessarily limited to presynaptic regions between hair cells and SGNs. A combined approach using genomics and neuroimaging in *OTOF*-associated ANSD subjects may set the stage for the optimal strategy to enhance language development.

## References

[1] Berlin CI, Hood LJ, Morlet T, Wilensky D, Li L, Mattingly KR, et al. Multi-site diagnosis and management of 260 patients with auditory neuropathy/dys-synchrony (auditory neuropathy spectrum disorder*). International journal of audiology. 2010;49(1):30–43. doi: 10.3109/14992020903160892 20053155

[2] Teagle HF, Roush PA, Woodard JS, Hatch DR, Zdanski CJ, Buss E, et al. Cochlear implantation in children with auditory neuropathy spectrum disorder. Ear and hearing. 2010;31(3):325–35. doi: 10.1097/AUD.0b013e3181ce693b 20090530

[3] Starr A. The neurology of auditory neuropathy. Sininger I, Starr A Auditory neuropathy, a new perspective on hearing disorders San Diego: Singular Publishing Group. 2001:37–49.

[4] Hassan DM. Perception of temporally modified speech in auditory neuropathy. International journal of audiology. 2011;50(1):41–9. doi: 10.3109/14992027.2010.520035 21047293

[5] Kim SH, Choi HS, Han YE, Choi BY. Diverse etiologies manifesting auditory neuropathy characteristics from infants with profound hearing loss and clinical implications. International journal of pediatric otorhinolaryngology. 2016;86:63–7. doi: 10.1016/j.ijporl.2016.04.013 27260582

[6] Kim BJ, Jang JH, Han JH, Park H-R, Oh DY, Lee S, et al. Mutational and phenotypic spectrum of OTOF-related auditory neuropathy in Koreans: eliciting reciprocal interaction between bench and clinics. Journal of translational medicine. 2018;16(1):330. doi: 10.1186/s12967-018-1708-z 30482216 PMC6260760

[7] Manchaiah VK, Zhao F, Danesh AA, Duprey R. The genetic basis of auditory neuropathy spectrum disorder (ANSD). International journal of pediatric otorhinolaryngology. 2011;75(2):151–8. doi: 10.1016/j.ijporl.2010.11.023 21176974

[8] Choi BY, Ahmed ZM, Riazuddin S, Bhinder M, Shahzad M, Husnain T, et al. Identities and frequencies of mutations of the otoferlin gene (OTOF) causing DFNB9 deafness in Pakistan. Clinical genetics. 2009;75(3):237–43. doi: 10.1111/j.1399-0004.2008.01128.x 19250381 PMC3461579

[9] Kim BJ, Jang JH, Han JH, Park H-R, Oh DY, Lee S, et al. Mutational and phenotypic spectrum of OTOF-related auditory neuropathy in Koreans: eliciting reciprocal interaction between bench and clinics. Journal of translational medicine. 2018;16(1):1–13. 30482216 10.1186/s12967-018-1708-z PMC6260760

[10] Jin YJ, Park J, Kim AR, Rah YC, Choi BY. Identification of a novel splice site variant of OTOF in the Korean nonsyndromic hearing loss population with low prevalence of the OTOF mutations. International journal of pediatric otorhinolaryngology. 2014;78(7):1030–5. doi: 10.1016/j.ijporl.2014.03.033 24814232

[11] Roux I, Safieddine S, Nouvian R, Grati Mh, Simmler M-C, Bahloul A, et al. Otoferlin, defective in a human deafness form, is essential for exocytosis at the auditory ribbon synapse. Cell. 2006;127(2):277–89. doi: 10.1016/j.cell.2006.08.040 17055430

[12] Pangršič T, Lasarow L, Reuter K, Takago H, Schwander M, Riedel D, et al. Hearing requires otoferlin-dependent efficient replenishment of synaptic vesicles in hair cells. Nature neuroscience. 2010;13(7):869. doi: 10.1038/nn.2578 20562868

[13] Michalski N, Goutman JD, Auclair SM, de Monvel JB, Tertrais M, Emptoz A, et al. Otoferlin acts as a Ca2+ sensor for vesicle fusion and vesicle pool replenishment at auditory hair cell ribbon synapses. Elife. 2017;6:e31013. doi: 10.7554/eLife.31013 29111973 PMC5700815

[14] Park JH, Kim AR, Han JH, Kim SD, Kim SH, Koo J-W, et al. Outcome of cochlear implantation in prelingually deafened children according to molecular genetic etiology. Ear and hearing. 2017;38(5):e316–e24. doi: 10.1097/AUD.0000000000000437 28841141

[15] Wunderlich JL, Cone-Wesson BK, Shepherd R. Maturation of the cortical auditory evoked potential in infants and young children. Hearing research. 2006;212(1–2):185–202. doi: 10.1016/j.heares.2005.11.010 16459037

[16] Sharma A, Dorman MF, Spahr AJ. A sensitive period for the development of the central auditory system in children with cochlear implants: implications for age of implantation. Ear and hearing. 2002;23(6):532–9. doi: 10.1097/00003446-200212000-00004 12476090

[17] Cardon G, Sharma A. Central auditory maturation and behavioral outcome in children with auditory neuropathy spectrum disorder who use cochlear implants. International journal of audiology. 2013;52(9):577–86. doi: 10.3109/14992027.2013.799786 23819618 PMC3781925

[18] Sharma A, Cardon G, Henion K, Roland P. Cortical maturation and behavioral outcomes in children with auditory neuropathy spectrum disorder. International journal of audiology. 2011;50(2):98–106. doi: 10.3109/14992027.2010.542492 21265637 PMC3735347

[19] Hood LJ, editor Variation in auditory neuropathy spectrum disorder: Implications for evaluation and management. Seminars in Hearing; 2011: © Thieme Medical Publishers.

[20] Chang MY, Kim AR, Kim NK, Lee C, Park W-Y, Choi BY. Refinement of molecular diagnostic protocol of auditory neuropathy Spectrum disorder: disclosure of significant level of etiologic homogeneity in Koreans and its clinical implications. Medicine. 2015;94(47). doi: 10.1097/MD.0000000000001996 26632695 PMC5058964

[21] Hosoya M, Minami SB, Enomoto C, Matsunaga T, Kaga K. Elongated EABR wave latencies observed in patients with auditory neuropathy caused by OTOF mutation. Laryngoscope investigative otolaryngology. 2018;3(5):388–93. doi: 10.1002/lio2.210 30410993 PMC6209615

[22] Żak M, Pfister M, Blin N. The otoferlin interactome in neurosensory hair cells: significance for synaptic vesicle release and trans-Golgi network. International journal of molecular medicine. 2011;28(3):311–4. doi: 10.3892/ijmm.2011.716 21643623

[23] Lee S-Y, Oh D-Y, Han JH, Kim MY, Kim B, Kim BJ, et al. Flexible Real-Time Polymerase Chain Reaction-Based Platforms for Detecting Deafness Mutations in Koreans: A Proposed Guideline for the Etiologic Diagnosis of Auditory Neuropathy Spectrum Disorder. Diagnostics. 2020;10(9):672. doi: 10.3390/diagnostics10090672 32899707 PMC7554951

[24] Bae S-H, Baek J-I, Lee JD, Song MH, Kwon T-J, Oh S-K, et al. Genetic analysis of auditory neuropathy spectrum disorder in the Korean population. Gene. 2013;522(1):65–9. doi: 10.1016/j.gene.2013.02.057 23562982

[25] Lee SY, Han JH, Kim BJ, Oh SH, Lee S, Oh DY, et al. Identification of a Potential Founder Effect of a Novel PDZD7 Variant Involved in Moderate-to-Severe Sensorineural Hearing Loss in Koreans. Int J Mol Sci. 2019;20(17). doi: 10.3390/ijms20174174 . 31454969 PMC6747409

[26] Lee SY, Joo K, Oh J, Han JH, Park HR, Lee S, et al. Severe or Profound Sensorineural Hearing Loss Caused by Novel USH2A Variants in Korea: Potential Genotype-Phenotype Correlation. Clin Exp Otorhinolaryngol. 2019. doi: 10.21053/ceo.2019.00990 . 31674169 PMC7248602

[27] Campbell JD, Cardon G, Sharma A, editors. Clinical application of the P1 cortical auditory evoked potential biomarker in children with sensorineural hearing loss and auditory neuropathy spectrum disorder. Seminars in hearing; 2011: NIH Public Access. 10.1055/s-0031-1277236 PMC3782746 24078765

[28] Sharma A, Glick H, Campbell J, Torres J, Dorman M, Zeitler DM. Cortical Plasticity and Reorganization in Pediatric Single-sided Deafness Pre- and Postcochlear Implantation: A Case Study. Otol Neurotol. 2016;37(2):e26–34. doi: 10.1097/MAO.0000000000000904 . 26756152 PMC6530986

[29] Kraus N, McGee TJ, Carrell TD, Zecker SG, Nicol TG, Koch DB. Auditory neurophysiologic responses and discrimination deficits in children with learning problems. Science. 1996;273(5277):971–3. doi: 10.1126/science.273.5277.971 . 8688085

[30] Bidelman GM, Pousson M, Dugas C, Fehrenbach A. Test-Retest Reliability of Dual-Recorded Brainstem versus Cortical Auditory-Evoked Potentials to Speech. Journal of the American Academy of Audiology. 2018;29(2):164–74. doi: 10.3766/jaaa.16167 29401063

[31] British Society of Audiology. Recommended procedure—cortical auditory evoked potential (CAEP) testing; 2016.

[32] Gilley PM, Sharma A, Dorman M, Finley CC, Panch AS, Martin K. Minimization of cochlear implant stimulus artifact in cortical auditory evoked potentials. Clin Neurophysiol. 2006;117(8):1772–82. doi: 10.1016/j.clinph.2006.04.018 . 16807102

[33] Oostenveld R, Fries P, Maris E, Schoffelen JM. FieldTrip: Open source software for advanced analysis of MEG, EEG, and invasive electrophysiological data. Comput Intell Neurosci. 2011;2011:156869. doi: 10.1155/2011/156869 . 21253357 PMC3021840

[34] Delorme A, Makeig S. EEGLAB: an open source toolbox for analysis of single-trial EEG dynamics including independent component analysis. J Neurosci Methods. 2004;134(1):9–21. doi: 10.1016/j.jneumeth.2003.10.009 . 15102499

[35] Dorman MF, Sharma A, Gilley P, Martin K, Roland P. Central auditory development: evidence from CAEP measurements in children fit with cochlear implants. J Commun Disord. 2007;40(4):284–94. doi: 10.1016/j.jcomdis.2007.03.007 . 17433357 PMC2755241

[36] Sharma A, Dorman MF, Kral A. The influence of a sensitive period on central auditory development in children with unilateral and bilateral cochlear implants. Hear Res. 2005;203(1–2):134–43. doi: 10.1016/j.heares.2004.12.010 . 15855038

[37] Sharma A, Kraus N, McGee TJ, Nicol TG. Developmental changes in P1 and N1 central auditory responses elicited by consonant-vowel syllables. Electroencephalogr Clin Neurophysiol. 1997;104(6):540–5. doi: 10.1016/s0168-5597(97)00050-6 . 9402896

[38] Sharma A, Dorman MF, Spahr AJ. Rapid development of cortical auditory evoked potentials after early cochlear implantation. Neuroreport. 2002;13(10):1365–8. doi: 10.1097/00001756-200207190-00030 12151804

[39] Dorman MF, Sharma A, Gilley P, Martin K, Roland P. Central auditory development: evidence from CAEP measurements in children fit with cochlear implants. Journal of communication disorders. 2007;40(4):284–94. doi: 10.1016/j.jcomdis.2007.03.007 17433357 PMC2755241

[40] Campbell J, Cardon G, Sharma A, editors. Clinical application of the P1 cortical auditory evoked potential biomarker in children with sensorineural hearing loss and auditory neuropathy spectrum disorder. Seminars in hearing; 2011: © Thieme Medical Publishers. 10.1055/s-0031-1277236 PMC3782746 24078765

[41] Cardon G, Campbell J, Sharma A. Plasticity in the developing auditory cortex: evidence from children with sensorineural hearing loss and auditory neuropathy spectrum disorder. Journal of the American Academy of Audiology. 2012;23(6):396–411. doi: 10.3766/jaaa.23.6.3 22668761 PMC3733172

[42] Sharma A, Cardon G. Cortical development and neuroplasticity in Auditory Neuropathy Spectrum Disorder. Hear Res. 2015;330(Pt B):221–32. doi: 10.1016/j.heares.2015.06.001 . 26070426 PMC4675684

[43] Emami SF, Abdoli A. Cortical Auditory Evoked Potentials in Children with Auditory Neuropathy/Dys-Synchrony. Indian Journal of Otolaryngology and Head & Neck Surgery. 2019;71(2):238–42. 31275837 10.1007/s12070-018-1445-x PMC6582116

[44] Pantelemon C, Necula V, Popa LL, Palade S, Strilciuc S, Muresanu DF. The Potential Use of P1 CAEP as a Biomarker for Assessing Central Auditory Pathway Maturation in Hearing loss and Associated Disabilities: a case report. Journal of Medicine and Life. 2019;12(4):457. doi: 10.25122/jml-2019-0096 32025267 PMC6993302

[45] Cardon G, Sharma A. Somatosensory Cross-Modal Reorganization in Children With Cochlear Implants. Frontiers in Neuroscience. 2019;13. doi: 10.3389/fnins.2019.00469 31312115 PMC6613479

[46] Lee S-Y, Shim YJ, Han J-H, Song J-J, Koo J-W, Oh SH, et al. the molecular etiology of deafness and auditory performance in the postlingually deafened cochlear implantees. Scientific reports. 2020;10(1):1–12. 32238869 10.1038/s41598-020-62647-y PMC7113281

[47] Wu C-M, Ko H-C, Tsou Y-T, Lin Y-H, Lin J-L, Chen C-K, et al. Long-term cochlear implant outcomes in children with GJB2 and SLC26A4 mutations. PloS one. 2015;10(9). doi: 10.1371/journal.pone.0138575 26397989 PMC4580418

[48] Roush P, Frymark T, Venediktov R, Wang B. Audiologic management of auditory neuropathy spectrum disorder in children: a systematic review of the literature. American Journal of Audiology. 2011. doi: 10.1044/1059-0889(2011/10-0032) 21940978

[49] Si Yasunaga, Mh Grati, Cohen-Salmon M, El-Amraoui A, Mustapha M, Salem N, et al. A mutation in OTOF, encoding otoferlin, a FER-1-like protein, causes DFNB9, a nonsyndromic form of deafness. Nature genetics. 1999;21(4):363–9. doi: 10.1038/7693 10192385

[50] Sharma A, Cardon G. Cortical development and neuroplasticity in auditory neuropathy spectrum disorder. Hearing research. 2015;330:221–32. doi: 10.1016/j.heares.2015.06.001 26070426 PMC4675684

[51] Pallas SL. Intrinsic and extrinsic factors that shape neocortical specification. Trends in neurosciences. 2001;24(7):417–23. doi: 10.1016/s0166-2236(00)01853-1 11410273

[52] Sanfins MD, Hatzopoulos S, Skarzynski PH, Donadon C. Neuroplasticity and the Auditory System. The Human Auditory System-Basic Features and Updates on Audiological Diagnosis and Therapy: IntechOpen; 2019.

[53] Persic D, Thomas ME, Pelekanos V, Ryugo DK, Takesian AE, Krumbholz K, et al. Regulation of auditory plasticity during critical periods and following hearing loss. Hearing Research. 2020:107976. doi: 10.1016/j.heares.2020.107976 32591097 PMC8546402

[54] Paulina P, Bartosz K, Małgorzata G, Katarzyna C, Rafał M, Agnieszka P, et al. Early general development and central auditory system maturation in children with cochlear implants–A case series. International journal of pediatric otorhinolaryngology. 2019;126:109625. 10.1016/j.ijporl.2019.109625 31442872

